# Intra‐trophic isotopic discrimination of ^15^N/^14^N for amino acids in autotrophs: Implications for nitrogen dynamics in ecological studies

**DOI:** 10.1002/ece3.2866

**Published:** 2017-03-22

**Authors:** Yuko Takizawa, Prarthana S. Dharampal, Shawn A. Steffan, Yoshinori Takano, Naohiko Ohkouchi, Yoshito Chikaraishi

**Affiliations:** ^1^ Faculty of Environmental Earth ScienceHokkaido UniversitySapporoJapan; ^2^ Institute of Low Temperature ScienceHokkaido UniversitySapporoJapan; ^3^ Japan Agency for Marine‐Earth Science and TechnologyYokosukaJapan; ^4^ Department of EntomologyUniversity of WisconsinMadisonWIUSA; ^5^ US Department of AgricultureAgricultural Research ServiceMadisonWIUSA

**Keywords:** food web, isotopic fractionation, plant phenology, trophic position, winter dormancy, δ^15^N

## Abstract

The differential discrimination of nitrogen isotopes (^15^N/^14^N) within amino acids in consumers and their diets has been routinely used to estimate organismal tropic position (TP). Analogous isotopic discrimination can occur within plants, particularly in organs lacking chloroplasts. Such discrimination likely arises from the catabolic deamination of amino acids, resulting in a numerical elevation of estimated TP, within newly synthesized biomass. To investigate this phenomenon, we examined the ^15^N/^14^N of amino acids (δ^15^ N_AA_) in spring leaves and flowers from eight deciduous and two annual plants. These plants were classified on the basis of their time of bloom, plants that bloomed when their leaves were absent (Type I) versus plants that bloomed while leaves were already present (Type II). Based on the δ^15^ N_AA_ values from leaves, both plant types occupied comparable and ecologically realistic mean TPs (=1.0 ± 0.1, mean ± 1σ). However, the estimated TPs of flowers varied significantly (Type I: 2.2 ± 0.2; Type II: 1.0 ± 0.1). We hypothesize that these results can be interpreted by the following sequence of events: (1) Type I floral biomass is synthesized in absence of active photosynthesis; (2) the catabolic deamination of amino acids in particular, leaves behind ^15^N in the residual pool of amino acids; and (3) the incorporation of these ^15^N‐enriched amino acids within the biomass of Type I flowers results in the numerical elevation of the TPs. In contrast, the actively photosynthesizing Type II leaves energetically sustain the synthesis of Type II flower biomass, precluding any reliance on catabolic deamination of amino acids. Amino acids within Type II flowers are therefore isotopically comparable to the Type II leaves. These findings demonstrate the idiosyncratic nature of the δ^15^ N_AA_ values within autotrophic organs and have implications for interpreting trophic hierarchies using primary producers and their consumers.

1

### 1.1 Introduction

Amino acid metabolism within consumers causes predictable enrichment in their ^15^N compared to their diets. By quantifying the magnitude of this isotopic discrimination among unique amino acids through compound‐specific isotope analysis (CSIA), ecologists have achieved unprecedented insights into complex food webs (e.g., Batista, Ravelo, Crusius, Casso, & McCarthy, 2014; Chikaraishi, Kashiyama, Ogawa, Kitazato, & Ohkouchi, 2007; Choy, Popp, Hannides, & Drazen, 2015; Dharampal & Findlay, 2017; Gaebler, Vitti, & Vukmirovich, 1966; McCarthy, Benner, Lee, & Fogel, 2007; McClelland & Montoya, 2002; Naito et al., 2016; Popp et al., 2007; Sackett, Drazen, Choy, Popp, & Pitz, 2015; Sherwood, Iehmann, Schuber, Scott, & McCarthy, 2011; Steffan, Chikaraishi, Currie, et al., 2015). Chikaraishi et al. (2009) measured this "inter"‐trophic isotopic discrimination between consumers and their diets and established the following equation (1) to estimate the trophic position (TP_Tr/Src_) of organisms in food webs:

(1) $${\text{TP}}_{\mathrm{Tr}/\mathrm{Src}}=\left(\frac{\delta^{15}N_{\mathrm{Tr}}-\delta^{15}N_{\mathrm{Src}}-\beta_{\mathrm{Tr}/\mathrm{Src}}}{{\text{TDF}}_{\mathrm{Tr}/\mathrm{Src}}}\right)+1$$

where δ^15^N_Tr_ and δ^15^N_Src_ denote stable nitrogen isotopic composition of trophic (Tr, including alanine, valine, isoleucine, proline, and glutamic acid) and source (Src, including methionine and phenylalanine) amino acids in a single organism examined, respectively; β_Tr/Src_ denotes the isotopic difference between Tr and Src amino acids in primary producers at the base of food webs; and TDF_Tr/Src_ (=Δδ^15^N_Tr_ − Δδ^15^N_Src_) stands for the net intertrophic discrimination factor of Tr and Src amino acids between a consumer and its diet. By investigating several pairs of Tr and Src amino acids, Chikaraishi et al. (2009) identified glutamic acid and phenylalanine as the best combination to return the most accurate estimation of the trophic position of consumers (TP_Glu/Phe_). Since then, several studies further suggested that using the average δ^15^N values of Tr and Src amino acids of multiple amino acids may provide greater statistical power to TP calculations than a single pair of amino acids (e.g., Bradley et al., 2015; Décima, Landry, & Popp, 2013; Nielsen, Popp, & Winder, 2015; Sherwood et al., 2011).

The unique metabolic pathway of individual amino acids can affect their isotopic behavior (whether Tr or Scr amino acids). Within heterotrophs, these differential enrichment (or depletion) patterns determine the amount of intertrophic isotopic discrimination (e.g., Chikaraishi et al., 2007, 2009; Ohkouchi, Ogawa, Chikaraishi, Tanaka, & Wada, 2015). For instance, it has been proposed that catabolic deamination (preceding transamination) of Tr amino acids causes the preferential cleavage of the ^14^N amino group, resulting in an accumulation of ^15^N (by up to ~3–8‰ per trophic level) in the Tr amino acids of consumer (Chikaraishi et al., 2007). In in vitro trials, Miura and Goto (2012) reported that the magnitude of isotopic discrimination of glutamic acid strongly correlates with its deamination flux (i.e., the deamination of a large pool generates greater isotopic discrimination compared to that from a smaller pool). However, the metabolism of Src amino acids does not involve the formation or cleaving of carbon–nitrogen bonds. Therefore, there is negligible isotopic discrimination in the Src amino acids between consumer and diet (Chikaraishi et al., 2007). The metabolic routing of amino acids may invoke alternative patterns of isotopic discrimination, particularly in the carbon isotopes of nonessential amino acids (McMahon, Fogel, Elsdon, & Thorrold, 2010). Although discrimination in nitrogen isotopes associated with metabolic routing has not been evidenced (Chikaraishi et al., 2007), the balance of amino acids, lipids, and carbohydrates as metabolic energy sources can potentially cause a significant variation in the isotopic discrimination of amino acids (Blanke et al., 2017; Chikaraishi, Steffan, Takano, & Ohkouchi, 2015; McMahon, Thorrold, Elsdon, & McCarthy, 2015).

With the exception of photosynthesis, there are several metabolic parallels between plants and heterotrophs (Figure 1, cf: Buchanan, Gruissem, & Jones, 2000). For example, plants can store photosynthetically fixed energy in form of carbohydrates, lipids, and/or amino acids (Buchanan et al., 2000; Chapin, Schulze, & Mooney, 1990; Kermode, 2011; Millard, 1996). During periods of lean photosynthesis, such as heterotrophs, the catabolism of these storage compounds releases energy (i.e., ATP) that is subsequently used for the anabolism of new constituents (Buchanan et al., 2000), particularly in organs without chloroplasts (e.g., flower and root). If this catabolism within autotrophic biomass involves the deamination of amino acids, the resulting residual pool of amino acids (particularly for Tr amino acids) will be more enriched in ^15^N than the original source pool. The mobilization and assimilation of these ^15^N‐enriched amino acids may generate isotopic differences between the source pool and newly synthesized biomass. Unlike "inter"‐trophic isotopic discrimination that involves two separate organisms with unique trophic identities (e.g., Chikaraishi et al., 2007), "intra"‐trophic isotopic discrimination arises as a result of catabolic deamination of storage amino acids among different tissues within a single plant. In almost all cases, photosynthetic energy fixation in plants exceeds catabolic energy release, even during limited availability of sunlight (Reich, Walters, Tjoelker, Vanderklein, & Buschena, 1998). The intratrophic isotopic discrimination in plants, primarily an outcome of amino acid deamination, is therefore hardly detectable during the growing season when metabolism is largely geared toward photosynthesis. The significant reduction or even absence of photosynthesis during winter dormancy, however, temporarily severs the energy supply for the homeostasis (Damesin, 2003). During this time, plants (mostly deciduous) must meet the energetic demands for the maintenance of basic cellular function through the catabolism of organic storage compounds (Arora, Wisniewski, & Scorza, 1992; Gomez & Faurobert, 2002; Loescher, McCamant, & Keller, 1990; Olofinboba, 1969), which may include deamination of storage amino acids, that ultimately results in the intratrophic isotopic discrimination within a plant tissue. Indeed, Takizawa and Chikaraishi (2014) first reported that sweet potato sprout grown in the absence of light has an unusually high TP_Glu/Phe_ value of 2.2. Given that sprouting occurred in dark, and in absence of photosynthesis, sprout biomass likely recorded the ^15^N‐enrichment derived from the amino acid deamination during catabolism.

**Figure 1:**
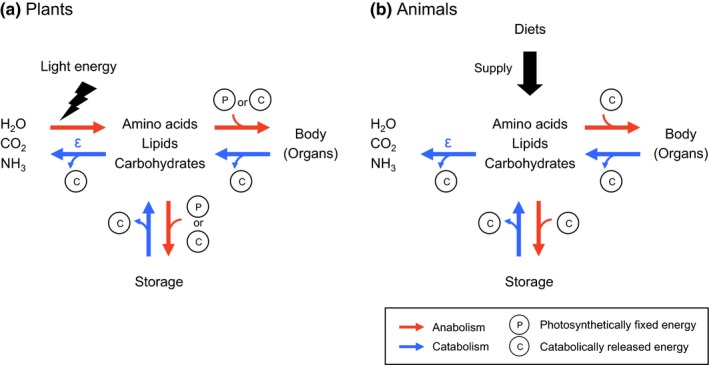
Schematic illustration of the catabolism and anabolism in (a) plants and (b) animals: The solar energy is fixed as organic molecules (e.g., amino acids, lipids, and carbohydrates) during photosynthesis in plants, and the fixed energy is released during metabolic breakdown of complex to simple molecules (i.e., catabolism) in both plants and animals; these energies are used during anabolism to construct of their body and storage biomass (after Buchanan et al., 2000)

We hypothesize that plant organs lacking chloroplasts may undergo intratrophic isotopic discrimination via the aforementioned mechanisms. This leads to an increased δ^15^N_Tr_ values in these organs, and therefore to an ecologically erroneous overestimation (TP_Glu/Phe_ > 1.0) for plant trophic position. The objective of our study was to investigate whether indeed there was a measurable amount of intratrophic isotopic discrimination between chloroplast‐bearing leaves and chloroplast‐lacking flowers, and to assess its implication for trophic position calculation.

### 1.2 Materials and Methods

#### 1.2.1 Leaf and flower samples

Flowers and mature leaves of eight deciduous trees and two annual plants were collected in their blooming season (February–May) from either a farm or a house garden in Yugawara, Japan (35°08′N, 139°07E) (Table S1). These plants commonly begin to grow leaves in spring and completely lose their leaves in autumn. They were classified into Type I and Type II, with respect to the timing of their bloom relative to leaf emergence (Figure 2). Type I plants included four stone fruit plants (*Amygdalus persica*,* Cerasus lannesiana*,* Cerasus pseudocerasus*, and *Prunus mume*) and one wisteria (*Wisteria floribunda*). These plants bloom for about 2–3 weeks prior to the emergence of their first leaves. Type II plants included three deciduous tree species (*Akebia quinata*,* Benthamidia japonica*, and *Hydrangea macrophylla*) and two annual plant species (*Cucumis sativus* and *Solanum melongena*). These plants bloom for 2–3 weeks (for deciduous plants), or continually for 1–2 months (for annual plants) only after their leaves have emerged. The flowers of the Type II plants were collected approximately halfway through the spring bloom. Both Type I and Type II plants were chosen as they are commonly found in agricultural area and/or house gardens in the temperate region of Japan. Approximately ten leaves and ten flowers were collected for each plant. The collected samples were cleaned with distilled water to remove surface contaminants, homogenized to a fine powder using a Tube‐Mill (IKA, Staufen, Germany), freeze‐dried, and then stored at −20°C.

**Figure 2:**
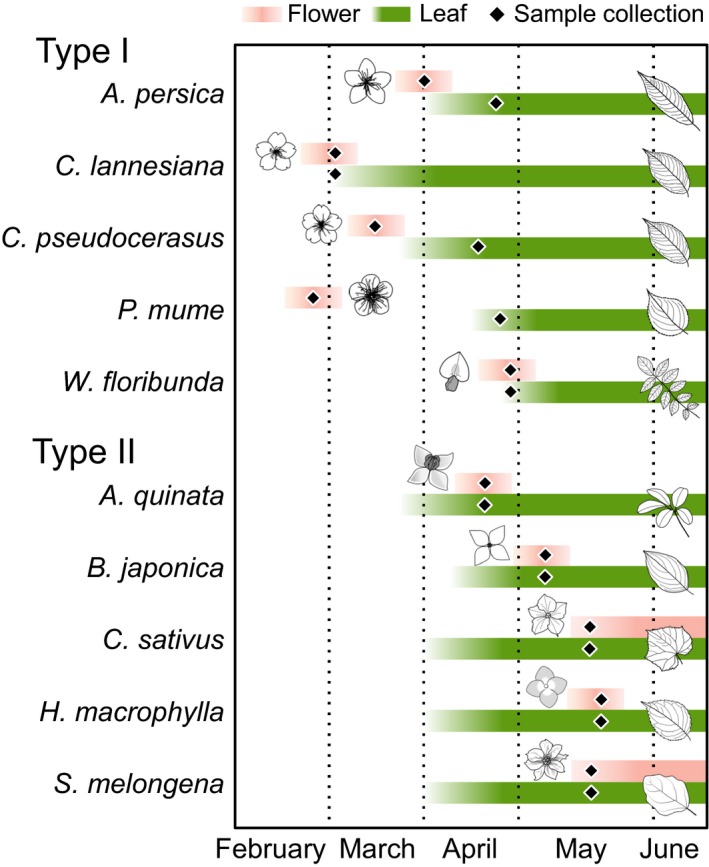
The periods of blooming and leafing for the plants induced in the present study. Flowers of the Type I plants (*Amygdalus persica*,* Cerasus lannesiana*,* Cerasus pseudocerasus*,* Prunus mume, and Wisteria floribunda*) bloomed for 2–3 weeks in the leafless period or just prior to the leafing period in spring. Flowers of the Type II plants bloomed for 2–3 weeks (for deciduous tree plants; *Akebia quinata*,* Benthamidia japonica*, and *Hydrangea macrophylla*) or continually for 1–2 months (for annual plants; *Cucumis sativus* and *Solanum melongena*) during the mature‐leaf period. Diamond symbols approximately indicate the collection dates (Table S1) of the flowers and leaves in the present study

#### 1.2.2 Analysis of the δ^15^N_AA_ values

The samples were prepared for the δ^15^N_AA_ analysis after HCl hydrolysis and *N*‐pivaloyl/isopropyl (Pv/iPr) derivatization, according to the procedure in Chikaraishi et al. (2009). In brief, the homogenized samples were hydrolyzed using 12 M HCl at 110°C overnight (>12 hr). The hydrolysate was washed with *n*‐hexane/dichloromethane (3/2, v/v) to remove hydrophobic constituents. The derivatization was performed sequentially with thionyl chloride/2‐propanol (1/4, v/v) at 110°C for 2 hr, and pivaloyl chloride/dichloromethane (1/4, v/v) at 110°C for 2 hr. The δ^15^N_AA_ values were determined by gas chromatography/isotope ratio mass spectrometry (GC/IRMS) using a 6890N GC (Agilent Technologies, Palo Alto, USA) instrument coupled to a Delta^plus^XP IRMS instrument through combustion (950°C) and reduction (550°C) furnaces, a countercurrent dryer (Permeable membrane, Nafion^™^), and a liquid nitrogen CO_2_ trap via a GC‐C/TC III interface (Thermo Fisher Scientific, Bremen, Germany). The Pv/iPr derivatives were injected using a programmable temperature vaporizing (PTV) injector (Gerstel, Mülheim, Germany) into an HP Ultra‐2 capillary column (50 m; i.d. 0.32 mm; film thickness 0.52 μm; Agilent Technologies). The carrier gas (He) flow rate was maintained at 1.4 ml/min. To assess the reproducibility of the isotope measurement, a standard amino acid reference mixture (Indiana University, Bloomington, USA; SI science co., Sugito‐machi, Japan) was analyzed after every five or six sample runs, with three pulses of reference N_2_ gas discharged at the beginning and end of each run. The δ^15^N_AA_ values were expressed relative to the isotopic composition of atmospheric nitrogen (AIR) on scales normalized to known δ^15^N values of the reference amino acids. The accuracy and precision for the reference mixtures were 0.0‰ (mean of ∆) and 0.3–0.5‰ (mean of 1σ) for sample sizes of ≥0.5 nmol N, respectively. The δ^15^N values of alanine, glycine, valine, leucine, isoleucine, proline, serine, glutamic acid, and phenylalanine were determined for sample leaves and flowers (Table 1), based on the S/N ratio of ≥20 with baseline separation on the chromatogram. All analyses were performed in triplicate, and the precision (1σ) for the δ^15^N_AA_ values in the sample amino acids was 0.0–0.8‰ (mean: 0.3 ± 0.2‰).

**Table 1:** Nitrogen isotopic composition of amino acids in plant leaves and flowers

Sample	δ^15^N (‰)^a^																		TP_Glu/Phe_ b		TDF′_Glu/Phe_ c
	Alanine		Glycine		Valine		Leucine		Isoleucine		Proline		Serine		Glutamic acid		Phenylalanine				
	Ave.	*SD*	Ave.	*SD*	Ave.	*SD*	Ave.	*SD*	Ave.	*SD*	Ave.	*SD*	Ave.	*SD*	Ave.	*SD*	Ave.	*SD*	*SD*		
Type I																					
Leaf																					
*Amygdalus persica*	−13.1	0.1	−16.7	0.2	−14.2	0.2	−13.1	0.1							−13.0	0.1	−5.1	0.1	1.1	0.02	
*Cerasus lannesiana*	−3.1	0.2	−13.0	0.4	−0.1	0.4	−4.2	0.2	−5.7	0.5	7.3	0.1			−0.7	0.4	6.9	0.3	1.1	0.09	
*Cerasus pseudocerasus*	−4.5	0.4	−17.4	0.5	−3.5	0.5	−5.7	0.4	−4.4	0.2	−0.2	0.2	−14.2	0.1	−2.1	0.3	6.3	0.3	1.0	0.03	
*Prunus mume*	3.5	0.4	−12.8	0.2	7.6	0.2	0.6	0.6	1.3	0.3	4.8	0.3			2.8	0.2	11.0	0.4	1.0	0.08	
*Wisteria floribunda*	2.1	0.6	1.1	0.8	3.8	0.3									5.1	0.3	13.3	0.6	1.0	0.12	
Flower																					
*Amygdalus persica*	−6.6	0.6	−15.2	0.3	−6.3	0.1							−10.0	0.4	−5.8	0.4	−4.0	0.1	1.9	0.08	6.5
*Cerasus lannesiana*	−1.7	0.1	−14.4	0.5	−0.3	0.2	−7.0	0.3	−2.8	0.3					1.4	0.4	0.6	0.4	2.2	0.00	9.2
*Cerasus pseudocerasus*	−3.3	0.3	−7.0	0.4	2.7	0.1	−1.2	0.4	−0.4	0.4	2.0	0.2			5.2	0.3	1.8	0.3	2.6	0.03	11.8
*Prunus mume*	−2.0	0.7	−8.2	0.7	0.5	0.6									7.3	0.3	5.8	0.3	2.3	0.06	9.9
*Wisteria floribunda*	−3.4	0.3	−14.3	0.5	−4.0	0.4	−9.1	0.7	−3.3	0.2			−15.3	0.3	0.2	0.6	−0.5	0.3	2.2	0.06	9.2
Type II																					
Leaf																					
*Akebia quinata*	−16.4	0.3	−25.6	0.2	−20.2	0.3	−17.5	0.2							−18.7	0.2	−10.0	0.1	1.0	0.02	
*Benthamidia japonica*	−12.1	0.2	−22.9	0.3	−10.4	0.4	−10.0	0.4							−12.4	0.7	−4.1	0.4	1.0	0.07	
*Cucumis sativus*	−1.3	0.5	−15.7	0.4	−2.1	0.2	−2.3	0.1	−2.7	0.4					0.3	0.4	8.9	0.3	1.0	0.09	
*Hydrangea macrophylla*	−3.7	0.4	−12.6	0.4	−2.1	0.2	−0.7	0.6	−1.7	0.0	−0.9	0.6	−11.0	0.0	−0.9	0.2	7.3	0.2	1.0	0.05	
*Solanum melongena*	−0.2	0.4	−18.4	0.4	0.5	0.5	0.6	0.2	2.5	0.2					2.4	0.6	11.8	0.3	0.9	0.11	
Flower																					
*Akebia quinata*			−24.9	0.3			−16.8	0.2							−16.6	0.5	−7.2	0.6	0.9	0.12	−0.9
*Benthamidia japonica*	−14.9	0.7	−30.8	0.5	−9.7	0.2									−13.2	0.2	−4.9	0.1	1.0	0.02	0.1
*Cucumis sativus*	−1.1	0.4	−16.6	0.1	−0.9	0.2	−1.2	0.1	−1.6	0.1					0.5	0.1	8.6	0.2	1.0	0.01	0.3
*Hydrangea macrophylla*	−3.1	0.5	−11.3	0.6	−1.4	0.3	−1.0	0.1	−0.5	0.4	1.0	0.3			0.1	0.3	8.3	0.3	1.0	0.07	0.2
*Solanum melongena*	−2.2	0.2	−14.9	0.0	−2.2	0.3	−4.5	0.1	−7.1	0.2					−1.0	0.2	7.0	0.4	1.1	0.07	0.4

a The δ^15^N value was determined by triplicate analysis for each sample.

b TP_Glu/Phe_ = [(δ^15^N_Glu_ − δ^15^N_Phe_ + 8.4)/7.6] + 1.

c TDF′_Glu/Phe_ = (δ^15^N_Flower,Glu_ − δ^15^N_Flower,Phe_) − β.

#### 1.2.3 Calculation of the TP_Glu/Phe_ values

The TP_Glu/Phe_ value was calculated from the observed δ^15^N values of glutamic acid (δ^15^N_Glu_) and phenylalanine (δ^15^N_Phe_), using equation (1) with the β_Glu/Phe_ and TDF_Glu/Phe_ (inter‐TDF_Glu/Phe_) being −8.4 ± 1.6‰ and +7.6 ± 1.2‰, respectively, values commonly applied for terrestrial samples from previous studies (Chikaraishi, Ogawa, Doi, & Ohkouchi, 2011; Chikaraishi, Ogawa, & Ohkouchi, 2010; Chikaraishi et al., 2014).

#### 1.2.4 Statistical analysis

Independent samples Mann–Whitney *U* test was used to compare the TP_Glu/Phe_ values of Type I leaves versus Type I flowers, and Type II leaves versus Type II flowers. Here, the null hypothesis was that TP_Glu/Phe_ values of leaves and flowers from any particular plant type would be indistinguishable. Independent samples Mann–Whitney *U* test was used to compare the TP_Glu/Phe_ values between Type I and Type II leaves, and independent samples *t* test was used to compare the TP_Glu/Phe_ values between Type I and Type II flowers. The null hypothesis assumed that the TP_Glu/Phe_ values of both leaves type would be comparable, as would both flower types. One‐sample Wilcoxon signed rank test was used to compare the TP_Glu/Phe_ values of Type I and Type II leaves, and one‐sample *t* test was used to compare Type I and Type II flowers to a test value of 1.0. The null hypothesis underlying these tests was that any plant sample whether leaves or flowers would have TP_Glu/Phe_ = 1.0.

### 1.3 Results and Discussion

#### 1.3.1 The δ^15^N_AA_ and TP_Glu/Phe_ values in leaves and flowers

Leaves and flowers fell within a similar but wide range in the δ^15^N_AA_ value within Type I and Type II plants (Type I leaves = −2.5 ± 8.2‰; Type I flowers = −3.5 ± 5.9‰ and Type II leaves = −6.3 ± 9.2‰; Type II flowers = −5.9 ± 9.2‰; mean ± 1σ, Table 1). As expected, TP_Glu/Phe_ value for both Type I and Type II leaves reported a mean of 1.0 ± 0.1 (Figure 3), consistent with previously reported values (1.0 ± 0.2) of plant samples such as leaves, nuts, and sap (Chikaraishi et al., 2011, 2014; Steffan et al., 2013). However, the TP_Glu/Phe_ value of Type I flowers (2.2 ± 0.2) was significantly higher than that of Type II flowers (1.0 ± 0.1) (*t*_8_ = 10.63, *p *<* *.001). Additionally, the TP_Glu/Phe_ value of Type I flowers was significantly higher than the functional trophic position of autotrophs (TP ~1.0) in any ecosystem (*t*_4_ = 11.05, *p *<* *.001). Conversely, the TP_Glu/Phe_ value of Type II flowers was virtually identical to the expected trophic position values (Figure 3). The TP_Glu/Phe_ value of Type I flowers was significantly higher than that of Type I leaves (TP_Flower_ − TP_Leaf_ = 1.2 ± 0.3; *U* = 25.0, *p* = .008). However, there was no such difference between the TP_Glu/Phe_ value of Type II flowers and leaves (TP_Flower_ − TP_Leaf_ = 0.0 ± 0.1).

**Figure 3:**
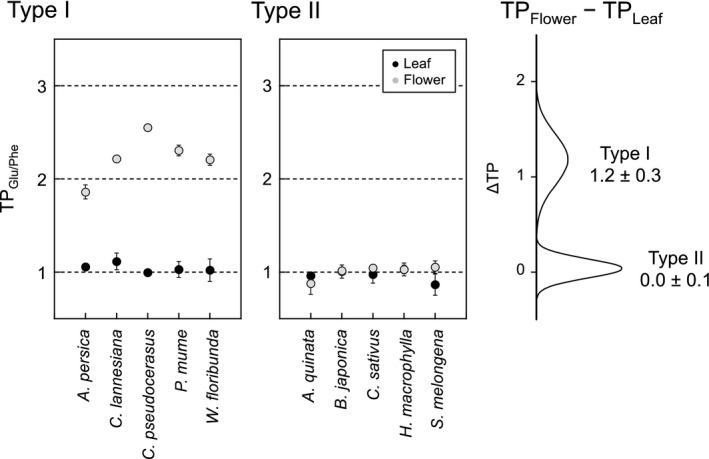
The TP_G__lu/Phe_ values of leaves and flowers from Type I and Type II plants, and the density distribution in the TP_G__lu/Phe_ value between leaves and flowers (ΔTP_F__lower−Leaf_) for these plants

#### 1.3.2 Energy resources for blooming

As primary producers, plants occupy TP = 1.0 (Elton, 1927; Lindeman, 1942), a value that has been validated using the CSIA method (Chikaraishi et al., 2011; McCarthy et al., 2013; Steffan et al., 2013). However, whether this well‐documented trophic identity applies to all organs within an individual plant has not been fully investigated (Takizawa & Chikaraishi, 2014). Our results indicate that even within a single plant, the Δδ^15^N_Glu−Phe_ value (and therefore the TP_Glu/Phe_ value) of different organs can vary significantly (Figures 3 and 4). We propose possible physiological scenarios that could contribute to this isotopic heterogeneity between leaves and flowers of Type I and Type II plants, potentially skewing their trophic identities.

**Figure 4:**
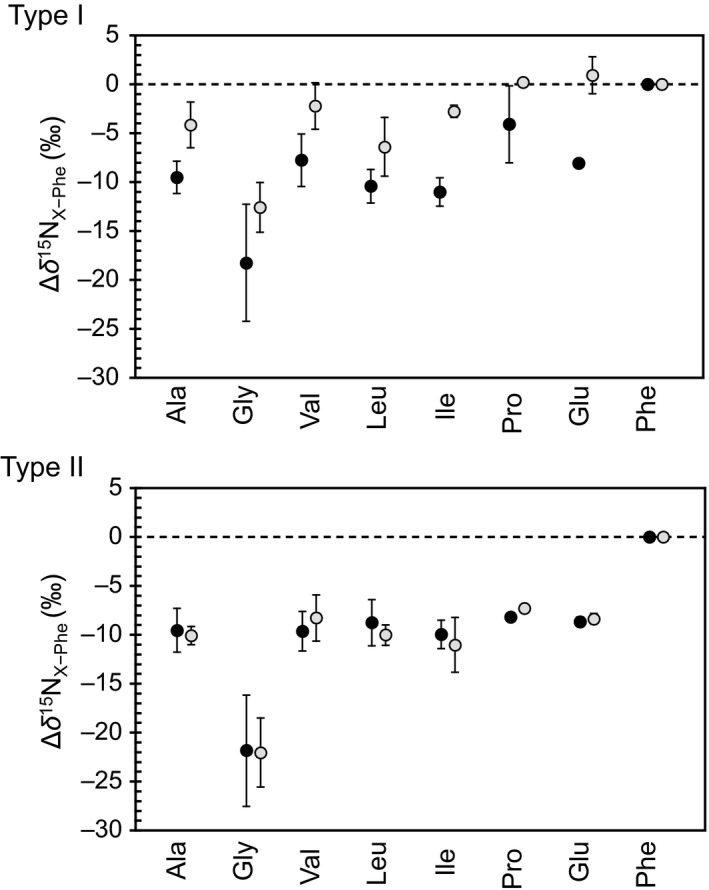
Difference in the δ^15^N value between amino acids and phenylalanine (Δδ^15^ N_X__−Phe_). Black‐ and gray‐filled circles indicate the value for leaves and flowers, respectively. Bar indicates 1σ variation within the plant types

Flowers have limited/no photosynthetic capacity and must therefore rely on other sources of energy to support their bloom. The elevation in the TP_Glu/Phe_ value (>1.0) in Type I flowers indicates that the Tr amino acids used to synthesize the flower biomass were enriched in ^15^N. One possible mechanism to explain this enrichment is that in Type I plants, bloom occurs before the appearance of leaves (i.e., in the absence of photosynthetically fixed energy). During this time, overwintered storage compounds are broken down to liberate metabolic energy (i.e., ATP) required to sustain Type I bloom. In case of stored proteins, the deamination of amino acids can preferentially eliminate the ^14^N amino group as ammonia, leaving behind the enriched ^15^N in the residual pool of Tr amino acids (Figure 5). If the enriched end products of deamination are used to assimilate Type I flowers, it would explain the enrichment of ^15^N in floral Tr amino acids. Previous studies have shown that antifreeze protein helps deciduous trees to survive through winter dormancy (Arora et al., 1992; Hon, Griffith, Mlynarz, Kwok, & Yang, 1995). Because these proteins are not required during spring, they may be subsequently deaminated (Arora et al., 1992). The residual pool of ^15^N‐enriched amino acids generates "intra"‐trophic discrimination, especially in Tr amino acids (e.g., glutamic acid), and when incorporated in newly synthesized Type I floral tissue, inflates their TP_Glu/Phe_ value (Figure 5).

**Figure 5:**
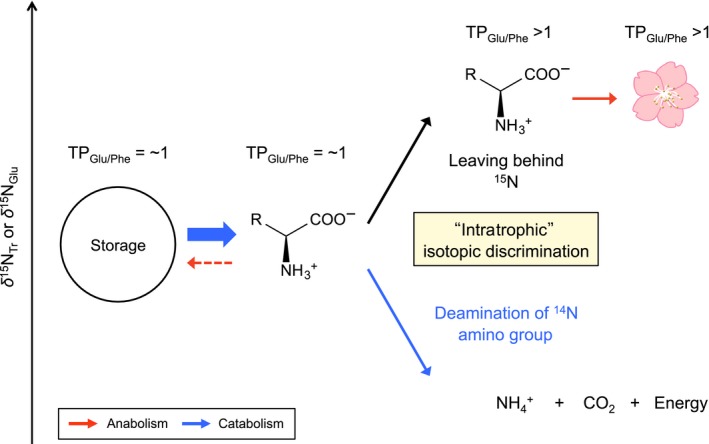
Possible metabolic states for flowering of the Type I plants, which includes deamination of amino acids and therefore alternative isotopic discrimination leading to significant elevation in the TP_G__lu/Phe_ value of amino acids in flowers

Type II plants represent a different phenology where bloom occurs in the presence of actively photosynthesizing leaves. As energy fixed by active photosynthesis is sufficient to support bloom, this may preclude the necessity of deamination of amino acids (Figure 5), which can explain the negligible intra‐trophic isotopic discrimination of amino acids in Type II plants. Therefore, the TP_Glu/Phe_ value of Type II leaves and Type II flowers remains comparable with each other, and to the expected value of 1.0.

#### 1.3.3 Intratrophic isotopic discrimination

We suggest the following equation (1) to illustrate the intra‐TDF of glutamic acid and phenylalanine (TDF′_Glu/Phe_) in plant organs (e.g., flowers):

(2) $${\text{TDF}}_{\mathrm{Glu}/\mathrm{Phe}}^{\prime }=\left(\delta^{15}N_{\mathrm{Organ},\mathrm{Glu}}-\delta^{15}N_{\mathrm{Organ},\mathrm{Phe}}\right)-\beta$$

where the subscript *Organ* indicates the plant organ of interest. The β should be derived from the δ^15^N value offset between glutamic acid and phenylalanine that has never undergone the deamination via plant catabolism. As our data show no substantial deamination in both Type I and Type II leaves, the standard β value (−8.4 ± 1.6‰, Chikaraishi et al., 2010) reported for primary producers was incorporated in equation (1).

Our data show that the TDF′_Glu/Phe_ for Type I flowers was 9.3 ± 1.9‰, elevating TP_Glu/Phe_ values of flowers correspondingly by 1.2 ± 0.3 trophic units than expected (Figure 3). This high TP_Glu/Phe_ value can be explained by a large TDF′_Glu/Phe_, probably as an outcome of the assimilation of deaminated amino acids. Type II flowers were most likely sustained by foliar photosynthesis during bloom, resulting in a low TDF′_Glu/Phe_ (0.0 ± 0.5‰), and a TP_Glu/Phe_ value ~1.0, as expected. Moreover, when equation (1) is applied to the previously reported data for sprout and sweet potato (TP_Glu/Phe_ = 2.2 and 1.4, respectively) (Takizawa & Chikaraishi, 2014), the TDF′_Glu/Phe_ is 9.1‰ for the sprout, and 2.8‰ for the sweet potato, indicative of strong reliance on deamination of amino acids for sprouting under dark conditions.

Interestingly, three of the five Type I plants (*A. persica*,* C. lannesiana*, and *W. floribunda*) included in this study started sprouting leaves from middle to end of the blooming period (Figure 2). It is plausible that these newly sprouted leaves are initially catabolically supported and later sustain themselves as photosynthesis ramps up. If so, the first few leaves to sprout could also present with higher TDF′, elevating their TP_Glu/Phe_ value temporarily, before returning to ecologically realistic values as the season progresses. Although we currently do not have sufficient data to support this assumption, further research is required to investigate intratrophic discrimination in leaves sprouting early in the season.

The TDF′_Glu/Phe_ will be a useful parameter to assess the magnitude of deamination of amino acids, with respect to the energy consumption in specific phenology, within plants. Several factors could result in variation of the TDF′_Glu/Phe_, including length of dormancy relative to growing season (short versus long), flower biomass relative to storage amino acids (small versus large), and availability of storage lipids and/or carbohydrates as alternative catabolic energy sources (low versus high). With further research, the TDF' parameter could potentially be extended and used to investigate unusually high trophic positions of herbivores (TP _Glu/Phe_ > 2.0).

#### 1.3.4 The δ^15^N values of phenylalanine in leaves and flowers

There was a large variation in the δ^15^N value of phenylalanine within Type I and Type II plants (Type I leaves = 6.5 ± 7.1‰; Type I flowers = 0.7 ± 3.6‰ and Type II leaves = 2.8 ± 9.3‰; Type II flowers = 2.3 ± 7.8‰), as well as a difference in phenylalanine value between leaves and flowers (Δδ^15^N_Phe_ = δ^15^N_Flower,Phe_ − δ^15^N_Leaf,Phe_) within a plant (Type I Δδ^15^N_Phe_ = −5.8 ± 5.3‰, and Type II Δδ^15^N_Phe_ = −0.4 ± 2.8‰). Such large variability in the δ^15^N_Phe_ and Δδ^15^N_Phe_ values is uncharacteristic, given that it is a Src amino acid. However, this variation is consistent with data published in previous studies using leaves collected from the same farm (10.6 ± 3.8‰) (Chikaraishi et al., 2011, 2014). One likely source of such variability is the temporal and spatial heterogeneity in the abundance and δ^15^N values of organic and inorganic nitrogen sources (NH_4_^+^, NO_3_^−^, and N_2_) in soils. The timing of bloom was different among the plants examined (Figure 2), and this could have introduced temporal variability in their δ^15^N values of phenylalanine. Additionally, the rate of incorporation of this isotopically variable phenylalanine may vary between leaves and flowers within a plant. However, evaluating the difference in the δ^15^N value between phenylalanine and other amino acids (Δδ^15^N_X−Phe_ =δ^15^N_X_ − δ^15^N_Phe_) can control for this background heterogeneity. Our data show that, notwithstanding this background noise, there was a significantly large difference between leaves and flowers for all examined amino acids in Type I plants (Δδ^15^N_X−Phe_ = 9.0 ± 2.1‰), but was negligible in Type II plants (Δδ^15^N_X−Phe_ = 0.3 ± 0.8‰) (Figure 4). Therefore, the background heterogeneity in the δ^15^N value of phenylalanine does not sufficiently explain the high Δδ^15^N_Phe_ value for Type I plants (Figure 4) and likely did not affect the numeral inflation of the TP_Glu/Phe_ values of Type I flowers (Figure 3). While our data show a clear pattern of differential isotopic discrimination in amino acids among plant tissues, we recommend further investigation using additional representative organs from Type I and Type II plants to evaluate the covariations in the TP_Glu/Phe_ value and TDF′_Glu/Phe_.

### 1.4 Implications

CSIA has expanded the ecologists’ toolbox by allowing high‐resolution insights into trophic interactions. However, little information is available about the factors controlling inter‐ and/or intratrophic isotopic discrimination of amino acids in plants, animals, fungi, and bacteria (Chikaraishi et al., 2015; Gutiérrez‐Rodríguez, Décima, Popp, & Landry, 2014; McMahon et al., 2015; Steffan, Chikaraishi, Currie, et al., 2015). Our findings reveal unique isotopic heterogeneity among wild plant organs, which can confound trophic estimations of these plants and the consumers that they support. For example, will feeding on Type I pollen in early spring elevate the TP_Glu/Phe_ of pollinators and nectarivores above that expected of herbivores (>2.0)? How will the preferential feeding of herbivores on early spring flowers or new leaves imprint on the trophic positions of higher order consumers? What will be the trophic identity of the detritus derived from Type I flowers as they become a basal resource in the brown food web?

Ideally, the TP_Glu/Phe_ (or TP_Tr/Src_) value enables isotopic ecologists to deduce the ecological function (e.g., primary producer, herbivore, omnivore, and carnivore) of organisms (Bradley et al., 2015; Chikaraishi et al., 2014; Nielsen et al., 2015; Steffan, Chikaraishi, Horton, et al., 2015). However, our data indicate that the TP_Glu/Phe_ value does not always reflect an organism's functional trophic position in the food web. For instance, although Type I flowers returned a TP_Glu/Phe_ value of 2.2, such value is typical of omnivores (TP > 2.0) and certainly does not represent the “functional” trophic identity of plants and their organs. Therefore, it appears that during sum of intra‐ and intertrophic isotopic discriminations, organismal TP_Glu/Phe_ values may represent “energetic” tendencies rather than the organism's true functional trophic position in food webs. These differences between the energetic and functional trophic positions arising from intratrophic isotopic discrimination can complicate food web studies. We therefore encourage continued investigations to reevaluate how CSIA‐derived trophic position correlates with the δ^15^N_AA_ values of organisms in food webs.

## Conflict of Interest

None declared.

## References

[ece32866-bib-0001] Arora, R. , Wisniewski, M. E. , & Scorza, R. (1992). Cold acclimation in genetically related (sibling) deciduous and evergreen peach (*Prunus persica* [L.] Batsch). Plant Physiology, 99, 1562–1568. 16669074 10.1104/pp.99.4.1562 PMC1080664

[ece32866-bib-0002] Batista, F. C. , Ravelo, A. C. , Crusius, J. , Casso, M. A. , & McCarthy, M. D. (2014). Compound specific amino acid δ^15^N in marine sediments: A new approach for studies of the marine nitrogen cycle. Geochimica et Cosmochimica Acta, 142, 553–569.

[ece32866-bib-0003] Blanke, C. , Chikaraishi, Y. , Takizawa, Y. , Steffan, S. , Dharampal, P. , & Vander Zanden, M. J. (2017). Comparing compound‐specific and bulk stable nitrogen isotope trophic discrimination factors across multiple freshwater fish species and diets. Canadian Journal of Fisheries and Aquatic Science. doi:10.1139/cjfas‐2016‐0420

[ece32866-bib-0004] Bradley, C. A. , Wallsgrove, N. J. , Choy, C. A. , Drazen, J. C. , Hetherington, E. D. , Hoen, D. K. , & Popp, B. N. (2015). Trophic position estimates of marine teleosts using amino acid compound specific isotopic analysis. Limnology and Oceanography Methods, 13, 476–493.

[ece32866-bib-0005] Buchanan, B. B. , Gruissem, W. , & Jones, R. L. (2000). Biochemistry and molecular biology of plants. Rockville, Maryland, USA: American Society of Plant Physiologists.

[ece32866-bib-0006] Chapin, F. S. I. I. I. , Schulze, E.‐D. , & Mooney, H. A. (1990). The ecology and economics of storage in plants. Annual Review of Ecology and Systematics, 21, 423–447.

[ece32866-bib-0007] Chikaraishi, Y. , Kashiyama, Y. , Ogawa, N. O. , Kitazato, H. , & Ohkouchi, N. (2007). Biosynthetic and metabolic controls of nitrogen isotopic composition of amino acids in marine macroalgae and gastropods: Implications for aquatic food web studies. Marine Ecology Progress Series, 342, 85–90.

[ece32866-bib-0008] Chikaraishi, Y. , Ogawa, N. O. , Doi, H. , & Ohkouchi, N. (2011). ^15^N/^14^N ratios of amino acids as a tool for studying terrestrial food webs: A case study of terrestrial insects (bees, wasps, and hornets). Ecological Research, 26, 835–844.

[ece32866-bib-0009] Chikaraishi, Y. , Ogawa, N. O. , Kashiyama, Y. , Takano, Y. , Suga, H. , Tomitani, A. , … Ohkouchi, N. (2009). Determination of aquatic food‐web structure based on compound‐specific nitrogen isotopic composition of amino acids. Limnology and Oceanography Methods, 7, 740–750.

[ece32866-bib-0010] Chikaraishi, Y. , Ogawa, N. O. , & Ohkouchi, N. (2010). Further evaluation of the trophic level estimation based on nitrogen isotopic composition of amino acids In Ohkouchi N., Tayasu I., & Koba K. (Eds.), Earth, life, and isotopes (pp. 37–51). Kyoto, Japan: Kyoto University Press.

[ece32866-bib-0011] Chikaraishi, Y. , Steffan, S. A. , Ogawa, N. O. , Ishikawa, N. F. , Sasaki, Y. , Tsuchiya, M. , & Ohkouchi, N. (2014). High‐resolution food webs based on nitrogen isotopic composition of amino acids. Ecology and Evolution, 4, 2423–2449. 25360278 10.1002/ece3.1103 PMC4203290

[ece32866-bib-0012] Chikaraishi, Y. , Steffan, S. A. , Takano, Y. , & Ohkouchi, N. (2015). Diet quality influences isotopic discrimination among amino acids in an aquatic vertebrate. Ecology and Evolution, 5, 2048–2059. 26045955 10.1002/ece3.1491 PMC4449758

[ece32866-bib-0013] Choy, C. A. , Popp, B. N. , Hannides, C. C. S. , & Drazen, J. C. (2015). Trophic structure and food resources of epipelagic and mesopelagic fishes in the North Pacific Subtropical Gyre ecosystem inferred from nitrogen isotopic compositions. Limnology and Oceanography, 60, 1156–1171.

[ece32866-bib-0014] Damesin, C. (2003). Respiration and photosynthesis characteristics of current‐year stems of *Fagus sylvatica*: From the seasonal pattern to an annual balance. New Phytologist, 158, 465–475. 10.1046/j.1469-8137.2003.00756.x 36056521

[ece32866-bib-0015] Décima, M. , Landry, M. R. , & Popp, B. N. (2013). Environmental perturbation effects on baseline ^15^N values and zooplankton trophic flexibility in the Southern California Current Ecosystem. Limnology and Oceanography, 58, 624–634.

[ece32866-bib-0016] Dharampal, P. S. , & Findlay, R. H. (2017). Mercury levels in largemouth bass (*Micropterus salmoides*) from regulated and unregulated rivers. Chemosphere, 170, 134–140. 27984776 10.1016/j.chemosphere.2016.11.162

[ece32866-bib-0017] Elton, C. S. (1927). Animal ecology. USA: The University Chicago Press.

[ece32866-bib-0018] Gaebler, O. H. , Vitti, T. G. , & Vukmirovich, R. (1966). Isotope effects in metabolism of ^14^N and ^15^N from unlabeled dietary proteins. Canadian Journal of Biochemistry, 44, 1249–1257. 6008218 10.1139/o66-142

[ece32866-bib-0019] Gomez, L. , & Faurobert, M. (2002). Contribution of vegetative storage proteins to seasonal nitrogen variations in the young shoots of peach trees (*Prunus persica* L. Batsch). Journal of Experimental Botany, 53, 2431–2439. 12432035 10.1093/jxb/erf098

[ece32866-bib-0020] Gutiérrez‐Rodríguez, A. , Décima, M. , Popp, B. N. , & Landry, M. R. (2014). Isotopic invisibility of protozoan trophic steps in marine food webs. Limnology and Oceanography, 59, 1590–1598.

[ece32866-bib-0021] Hon, W. H. , Griffith, M. , Mlynarz, A. , Kwok, Y. C. , & Yang, D. S. C. (1995). Antifreeze proteins in winter rye are similar to pathogenesis‐related proteins. Plant Physiology, 109, 879–889. 8552719 10.1104/pp.109.3.879 PMC161389

[ece32866-bib-0022] Kermode, A. R. (2011). Plant storage products (carbohydrates, oils and proteins). eLS. doi:10.1002/9780470015902.a0001325.pub2

[ece32866-bib-0501] Lindeman, R. L. (1942). The trophic‐dynamic aspect of ecology. Ecology, 23, 399–417.

[ece32866-bib-0023] Loescher, W. H. , McCamant, T. , & Keller, J. D. (1990). Carbohydrate reserves, translocation, and storage in woody plant roots. HortScience, 25, 274–281.

[ece32866-bib-0024] McCarthy, M. D. , Benner, R. , Lee, C. , & Fogel, M. L. (2007). Amino acid nitrogen isotopic fractionation patterns as indicators of heterotrophy in plankton, particulate, and dissolved organic matter. Geochimica et Cosmochimica Acta, 71, 4727–2744.

[ece32866-bib-0502] McCarthy, M. D. , Lehman, J. , & Kudela, R. (2013). Compound‐specific amino acid δ^15^N patterns in marine algae: Tracer potential for cyanobacterial vs. eukaryotic organic nitrogen sources in the ocean. Geochemica et Cosmochimica Acta, 103, 104–120.

[ece32866-bib-0025] McClelland, J. W. , & Montoya, J. P. (2002). Trophic relationships and the nitrogen isotopic composition of amino acids in plankton. Ecology, 83, 2173–2180.

[ece32866-bib-0026] McMahon, K. W. , Fogel, M. L. , Elsdon, T. S. , & Thorrold, S. R. (2010). Carbon isotope fractionation of amino acids in fish muscle reflects biosynthesis and isotopic routing from dietary protein. Journal of Animal Ecology, 79, 1132–1141. 20629794 10.1111/j.1365-2656.2010.01722.x

[ece32866-bib-0027] McMahon, K. W. , Thorrold, S. R. , Elsdon, T. S. , & McCarthy, M. D. (2015). Trophic discrimination of nitrogen stable isotopes in amino acids varies with diet quality in a marine fish. Limnology and Oceanography, 60, 1076–1087.

[ece32866-bib-0028] Millard, P. (1996). Ecophysiology of the internal cycling of nitrogen for tree growth. Journal of Plant Nutrition and Soil Science, 159, 1–10.

[ece32866-bib-0029] Miura, K. , & Goto, A. S. (2012). Stable nitrogen isotopic fractionation associated with transamination of glutamic acid to aspartic acid: Implications for understanding ^15^N trophic enrichment in ecological food webs. Researches in Organic Geochemistry, 28, 13–17.

[ece32866-bib-0030] Naito, Y. I. , Chikaraishi, Y. , Drucker, D. G. , Ohkouchi, N. , Semal, P. , Wißing, C. , & Bocherens, H. (2016). Ecological niche of Neanderthals from Spy Cave revealed by nitrogen isotopes of individual amino acids in collagen. Journal of Human Evolution, 93, 82–90. 27086057 10.1016/j.jhevol.2016.01.009

[ece32866-bib-0031] Nielsen, J. M. , Popp, B. N. , & Winder, M. (2015). Meta‐analysis of amino acid stable nitrogen isotope ratios for estimating trophic position in marine organisms. Oeologia, 178, 631–642. 10.1007/s00442-015-3305-7 25843809

[ece32866-bib-0032] Ohkouchi, N. , Ogawa, N. O. , Chikaraishi, Y. , Tanaka, H. , & Wada, E. (2015). Biochemical and physiological bases for the use of carbon and nitrogen isotopes in environmental and ecological studies. Progress in Earth and Planetary Science, 2, 1–17. doi:10.1186/s40645‐015‐0032‐y

[ece32866-bib-0033] Olofinboba, M. O. (1969). Seasonal variations in the carbohydrates in the xylem of *Antiaris Africana* . Annals of Botany, 33, 339–349.

[ece32866-bib-0034] Popp, B. N. , Graham, B. S. , Olson, R. J. , Hannides, C. C. S. , Lott, M. , López‐Ibarra, G. , & Galván‐Magaña, F. (2007). Insight into the trophic ecology of yellowfin tuna, *Thunnus albacares*, from compound‐specific nitrogen isotope analysis of proteinaceous amino acids In Dawson T. E., & Siegwolf R. T. W. (Eds.), Stable isotopes as indicators of ecological change (pp. 173–190). San Diego, USA: Academic Press.

[ece32866-bib-0035] Reich, P. B. , Walters, M. B. , Tjoelker, M. G. , Vanderklein, D. , & Buschena, C. (1998). Photosynthesis and respiration rates depend on leaf and root morphology and nitrogen concentration in nine boreal tree species differing in relative growth rate. Functional Ecology, 12, 395–405.

[ece32866-bib-0036] Sackett, D. K. , Drazen, J. C. , Choy, C. A. , Popp, B. , & Pitz, G. L. (2015). Mercury sources and trophic ecology for Hawaiian Bottomfish. Environmental Science and Technology, 49, 6909–6918. 25936419 10.1021/acs.est.5b01009

[ece32866-bib-0037] Sherwood, O. A. , Iehmann, M. F. , Schuber, C. J. , Scott, D. B. , & McCarthy, M. D. (2011). Nutrient regime shift in the western North Atlantic indicated by compound‐specific δ^15^N of deep‐sea gorgonian corals. Proceedings of the National Academy of Sciences of the United States of America, 108, 1011–1015. 21199952 10.1073/pnas.1004904108 PMC3024653

[ece32866-bib-0038] Steffan, S. A. , Chikaraishi, Y. , Currie, C. R. , Horn, H. , Gaines‐Day, H. R. , Pauli, J. N. , … Ohkouchi, N. (2015). Microbes are trophic analogs of animals. Proceedings of the National Academy of Sciences of the United States of America, 112, 15119–15124. 26598691 10.1073/pnas.1508782112 PMC4679051

[ece32866-bib-0039] Steffan, S. A. , Chikaraishi, Y. , Horton, D. R. , Miliczky, E. , Zalapa, J. E. , Jones, V. P. , & Ohkouchi, N. (2015). Beneficial or not? Decoding carnivore roles in plant protection. Biological Control, 91, 34–41.

[ece32866-bib-0040] Steffan, S. A. , Chikaraishi, Y. , Horton, D. R. , Ohkouchi, N. , Singleton, M. E. , Miliczky, E. , … Jones, V. P. (2013). Trophic hierarchies illuminated via amino acid isotopic analysis. PLoS ONE, 8, e76152. 24086703 10.1371/journal.pone.0076152 PMC3783375

[ece32866-bib-0041] Takizawa, Y. , & Chikaraishi, Y. (2014). Are baby sprouts eating the proteins in the mother sweet potato? Researches in Organic Geochemistry, 30, 29–32.

